# When is enough, enough? Understanding and solving your sample size problems in health services research

**DOI:** 10.1186/s13104-016-1893-x

**Published:** 2016-02-12

**Authors:** Victoria Pye, Natalie Taylor, Robyn Clay-Williams, Jeffrey Braithwaite

**Affiliations:** Australian Institute of Health Innovation, Faculty of Medicine and Health Sciences, Macquarie University, Level 6, 75 Talavera Road, Macquarie University, Sydney, NSW 2109 Australia

**Keywords:** Sample size, Effect size, Health services research, Methodologies

## Abstract

Health services researchers face two obstacles to sample size calculation: inaccessible, highly specialised or overly technical literature, and difficulty securing methodologists during the planning stages of research. The purpose of this article is to provide pragmatic sample size calculation guidance for researchers who are designing a health services study. We aimed to create a simplified and generalizable process for sample size calculation, by (1) summarising key factors and considerations in determining a sample size, (2) developing practical steps for researchers—illustrated by a case study and, (3) providing a list of resources to steer researchers to the next stage of their calculations. Health services researchers can use this guidance to improve their understanding of sample size calculation, and implement these steps in their research practice.

**Electronic supplementary material:**

The online version of this article (doi:10.1186/s13104-016-1893-x) contains supplementary material, which is available to authorized users.

## Findings

### Background

**Fig. 1:**
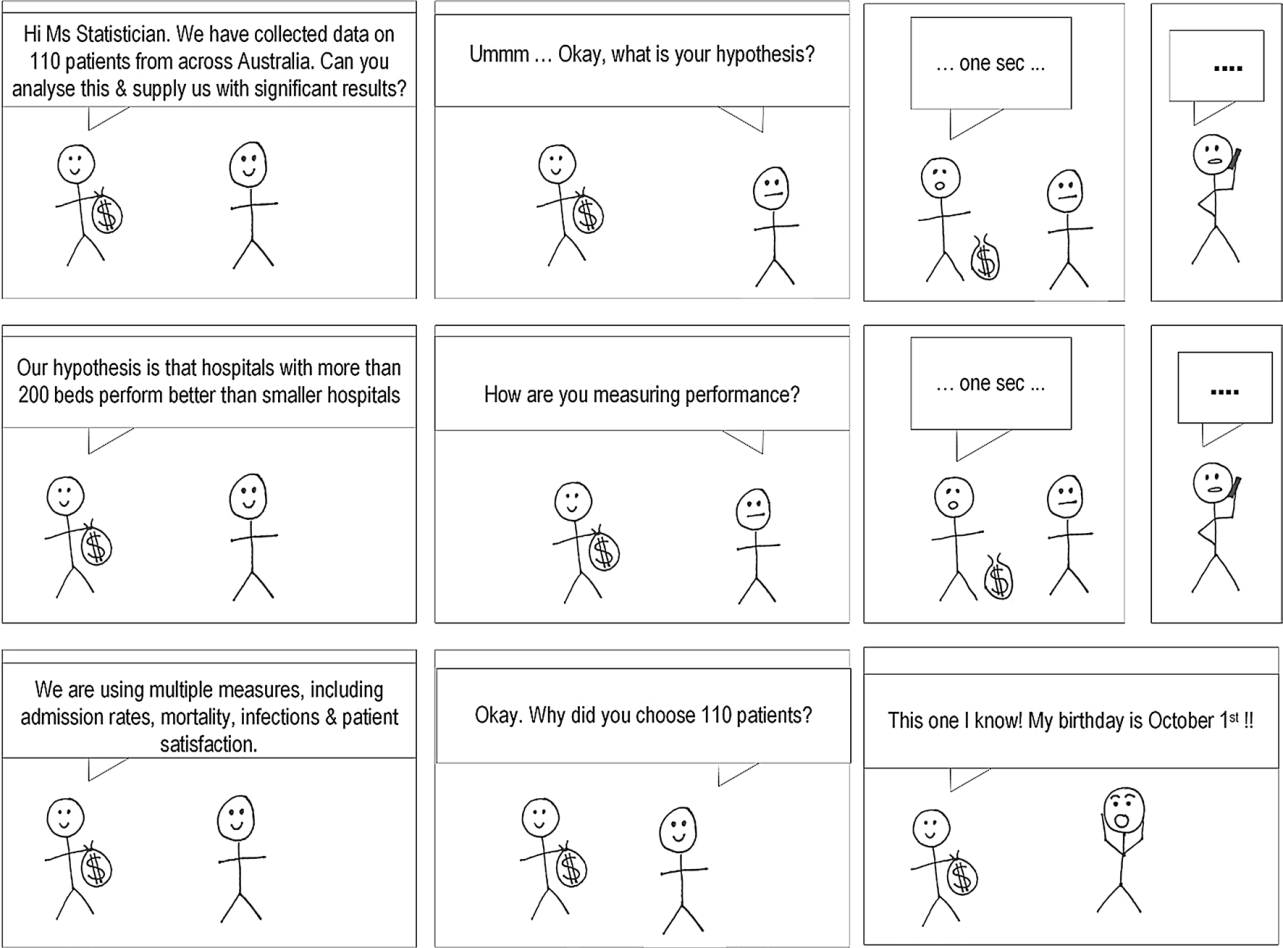
A statistician’s dilemma

Sample size literature for randomized controlled trials and study designs in which there is a clear hypothesis, single outcome measure, and simple comparison groups is available in abundance. Unfortunately health services research does not always fit into these constraints. Rather, it is often cross-sectional, and observational (i.e., with no ‘experimental group’) with multiple outcomes measured simultaneously. It can also be difficult work with no a priori hypothesis. The aim of this paper is to guide researchers during the planning stages to adequately power their study and to avoid the situation described in Fig. 1. By blending key pieces of methodological literature with a pragmatic approach, researchers will be equipped with valuable information to plan and conduct sufficiently powered research using appropriate methodological designs. A short case study is provided (Additional file 1) to illustrate how these methods can be applied in practice.

**Fig. 2:**
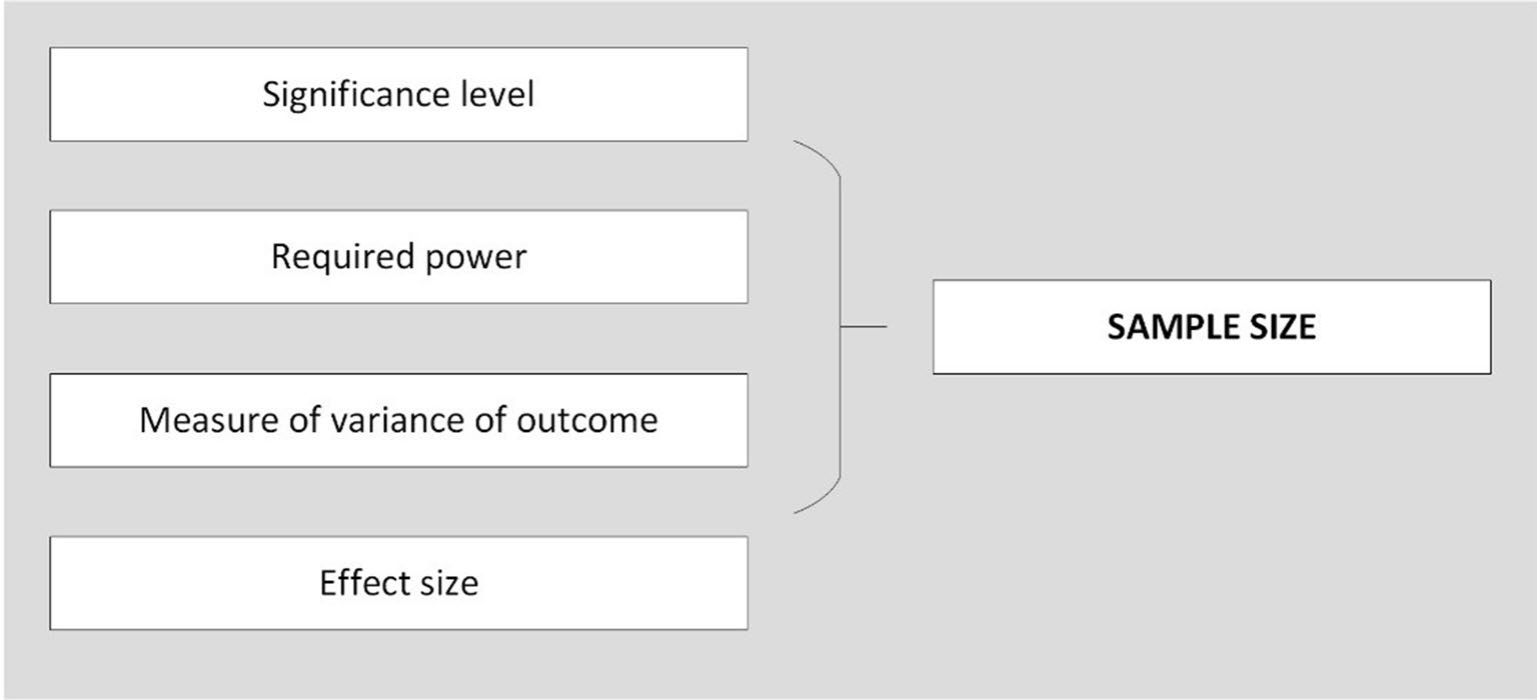
Inputs for a sample size calculation

The importance of an accurate sample size calculation when designing quantitative research is well documented [1–3]. Without a carefully considered calculation, results can be missed, biased or just plain incorrect. In addition to squandering precious research funds, the implications of a poor sample size calculation can render a study unethical, unpublishable, or both. For simple study designs undertaken in controlled settings, there is a wealth of evidence based guidance on sample size calculations for clinical trials, experimental studies, and various types of rigorous analyses (Table 1), which can help make this process relatively straightforward. Although experimental trials (e.g., testing new treatment methods) are undertaken within health care settings, research to further understand and improve the health service itself is often cross-sectional, involves no intervention, and is likely to be observing multiple associations [4]. For example, testing the association between leadership on hospital wards and patient re-admission, controlling for various factors such as ward speciality, size of team, and staff turnover, would likely involve collecting a variety of data (e.g., personal information, surveys, administrative data) at one time point, with no experimental group or single hypothesis. Multi-method study designs of this type create challenges, as inputs for an adequate sample size calculation are often not readily available. These inputs are typically: defined groups for comparison, a hypothesis about the difference in outcome between the groups (an effect size), an estimate of the distribution of the outcome (variance), and desired levels of significance and power to find these differences (Fig. 2).

**Table 1:** References for sample size calculation

Title	Primer	Concepts	Sample size	ROT	Simulation
Some practical guidelines for effective sample size determination [2]	✓	✓			
Sample size calculations for the design of health studies: a review of key concepts for non-statisticians [1]	✓	✓			
Sample size calculations: basic principles and common pitfalls [15]	✓	✓			
Sample size: how many participants do I need in my research? [3]	✓	✓			
Using effect size–or why the P value is not enough [8]	✓	✓			
Statistics and ethics: some advice for young statisticians [16]		✓			
Separated at birth: statisticians, social scientists and causality in health services research [17]		✓			
Reporting the results of epidemiological studies [9]		✓			
Surgical mortality as an indicator of hospital quality: the problem with small sample size [18]		✓			
Do multiple outcome measures require p-value adjustment? [11]		✓			
The problem of multiple inference in studies designed to generate hypothesis [19]		✓			
Understanding power and rules of thumb for determining sample sizes [20]		✓		✓	
Statistical rules of thumb [21]				✓	
A suggested statistical procedure for estimating the minimum sample size required for a complex cross-sectional study [22]			Complex cross-sectional		
A simple method of sample size calculation for liner and logistic regression [23]			Regression		✓
How many subjects does it take to do a regression analysis [10]			Regression		✓
Sample size determination in logistic regression [24]			Logistic regression		✓
A simulation study of the number of events per variable in a logistic regressions analysis [25]			Logistic regression		✓
Power and sample size calculations for studies involving linear regression [26]			Linear regression		✓
How to calculate sample size in randomized controlled trial? [27]			Randomised control trial		✓
Sufficient sample sizes for multilevel modelling [28]			Multilevel		✓
Sample size considerations for multilevel surveys [29]			Multilevel		✓
Sample size and accuracy of estimates in multilevel models: new simulation results [30]			Multilevel		✓
Robustness issues in multilevel regression analysis [31]			Multilevel		✓

Primer =  basic paper on the concepts around sample size determination, provides a basic but important understanding. Concepts =  provides a more detailed explanation around specific aspects of sample size calculation. Sample size =  these papers provide examples of sample size calculation for specific analysis types. ROT =  these papers provide sample size ‘rules of thumb’ for one or more type of analysis. Simulation =  these papers report the results of sample size simulation for various types of analysis

Even in large studies there is often an absence of funding for statistical support, or the funding is inadequate for the size of the project [5]. This is particularly evident in the planning phase, which is arguably when it is required the most [6]. A study by Altman et al. [7] of statistician involvement in 704 papers submitted to the *British Medical Journal* and *Annals of Internal Medicine* indicated that only 51 % of observational studies received input from trained biostatisticians and, even when accounting for contributions from epidemiologists and other methodologists, only 52 % of observational studies utilized statistical advice in the study planning phase [7]. The practice of health services researchers performing their own statistical analysis without appropriate training or consultation from trained statisticians is not considered ideal [5]. In the review decisions of journal editors, manuscripts describing studies requiring statistical expertise are more likely to be rejected prior to peer review if the contribution of a statistician or methodologist has not been declared [7].

Calculating an appropriate sample size is not only to be considered a means to an end in obtaining accurate results. It is an important part of planning research, which will shape the eventual study design and data collection processes. Attacking the problem of sample size is also a good way of testing the validity of the study, confirming the research questions and clarifying the research to be undertaken and the potential outcomes. After all it is unethical to conduct research that is knowingly either overpowered or underpowered [2, 3]. A study using more participants then necessary is a waste of resources and the time and effort of participants. An underpowered study is of limited benefit to the scientific community and is similarly wasteful.

With this in mind, it is surprising that methodologists such as statisticians are not customarily included in the study design phase. Whilst a lack of funding is partially to blame, it might also be that because sample size calculation and study design seem relatively simple on the surface, it is deemed unnecessary to enlist statistical expertise, or that it is only needed during the analysis phase. However, literature on sample size normally revolves around a single well defined hypothesis, an expected effect size, two groups to compare, and a known variance—an unlikely situation in practice, and a situation that can only occur with good planning. A well thought out study and analysis plan, formed in a conjunction with a statistician, can be utilized effectively and independently by researchers with the help of available literature. However a poorly planned study cannot be corrected by a statistician after the fact. For this reason a methodologist should be consulted early when designing the study.

Yet there is help if a statistician or methodologist is not available. The following steps provide useful information to aid researchers in designing their study and calculating sample size. Additionally, a list of resources (Table 1) that broadly frame sample size calculation is provided to guide researchers toward further literature searches.^1^

### A place to begin

Merrifield and Smith [1], and Martinez-Mesa et al. [3] discuss simple sample size calculations and explain the key concepts (e.g., power, effect size and significance) in simple terms and from a general health research perspective. These are a useful reference for non-statisticians and a good place to start for researchers who need a quick reminder of the basics. Lenth [2] provides an excellent and detailed exposition of effect size, including what one should avoid in sample size calculation.

**Fig. 3:**
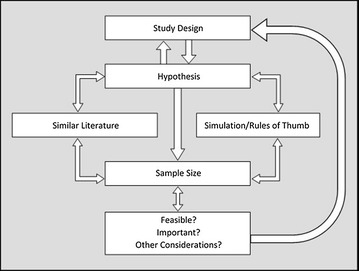
Stages in sample size calculation

Despite the guidance provided by this literature, there are additional factors to consider when determining sample size in health services research. Sample size requires deliberation from the outset of the study. Figure 3 depicts how different aspects of research are related to sample size and how each should be considered as part of an iterative planning phase. The components of this process are detailed below.

### Study design and hypothesis

The study design and hypothesis of a research project are two sides of the same coin. When there is a single unifying hypothesis, clear comparison groups and an effect size, e.g., drug A will reduce blood pressure 10 % more than drug B, then the study design becomes clear and the sample size can be calculated with relative ease. In this situation all the inputs are available for the diagram in Fig. 2.

However, in large scale or complex health services research the aim is often to further our understanding about the way the system works, and to inform the design of appropriate interventions for improvement. Data collected for this purpose is cross-sectional in nature, with multiple variables within health care (e.g., processes, perceptions, outputs, outcomes, costs) collected simultaneously to build an accurate picture of a complex system. It is unlikely that there is a single hypothesis that can be used for the sample size calculation, and in many cases much of the hypothesising may not be performed until after some initial descriptive analysis. So how does one move forward?

To begin, consider your hypothesis (one or multiple). What relationships do you want to find specifically? There are three reasons why you may not find the relationships you are looking for:

1. The relationship does not exist.
2. The study was not adequately powered to find the relationship.
3. The relationship was obscured by other relationships.

There is no way to avoid the first, avoiding the second involves a good understanding of power and effect size (see Lenth [2]), and avoiding the third requires an understanding of your data and your area of research. A sample size calculation needs to be well thought out so that the research can either find the relationship, or, if one is not found, to be clear why it wasn’t found. The problem remains that before an estimate of the effect size can be made, a single hypothesis, single outcome measure and study design is required. If there is more than one outcome measure, then each requires an independent sample size calculation as each outcome measure has a unique distribution. Even with an analysis approach confirmed (e.g., a multilevel model), it can be difficult to decide which effect size measure should be used if there is a lack of research evidence in the area, or a lack of consensus within the literature about which effect sizes are appropriate. For example, despite the fact that Lenth advises researchers to avoid using Cohen’s effect size measurements [2], these margins are regularly applied [8].

To overcome these challenges, the following processes are recommended:

1. Select a primary hypothesis. Although the study may aim to assess a large variety of outcomes and independent variables, it is useful to consider if there is one relationship that is of most importance. For example, for a study attempting to assess mortality, re-admissions and length of stay as outcomes, each outcome will require its own hypothesis. It may be that for this particular study, re-admission rates are most important, therefore the study should be powered first and foremost to address that hypothesis. Walker [9] describes why having a single hypothesis is easier to communicate and how the results for primary and secondary hypotheses should be reported.
2. Consider a set of important hypotheses and the ways in which you might have to answer each one. Each hypothesis will likely require different statistical tests and methods. Take the example of a study aiming to understand more about the factors associated with hospital outcomes through multiple tests for associations between outcomes such as length of stay, mortality, and readmission rates (dependent variables) and nurse experience, nurse-patient ratio and nurse satisfaction (independent variables). Each of these investigations may use a different type of analysis, a different statistical test, and have a unique sample size requirement. It would be possible to roughly calculate the requirements and select the largest one as the overall sample size for the study. This way, the tests that require smaller samples are sure to be adequately powered. This option requires more time and understanding than the first.

### Literature

During the study planning phase, when a literature review is normally undertaken, it is important not only to assess the findings of previous research, but also the design and the analysis. During the literature review phase, it is useful to keep a record of the study designs, outcome measures, and sample sizes that have already been reported. Consider whether those studies were adequately powered by examining the standard errors of the results and note any reported variances of outcome variables that are likely to be measured.

One of the most difficult challenges is to establish an appropriate expected effect size. This is often not available in the literature and has to be a judgement call based on experience. However previous studies may provide insight into clinically significant differences and the distribution of outcome measures, which can be used to help determine the effect size. It is recommended that experts in the research area are consulted to inform the decision about the expected effect size [2, 8].

### Simulation and rules of thumb

For many study designs, simulation studies are available (Table 1). Simulation studies generally perform multiple simulated experiments on fictional data using different effect sizes, outcomes and sample sizes. From this, an estimation of the standard error and any bias can be identified for the different conditions of the experiments. These are great tools and provide ‘ball park’ figures for similar (although most likely not identical) study designs. As evident in Table 1, simulation studies often accompany discussions of sample size calculations. Simulation studies also provide ‘rules of thumb’, or heuristics about certain study designs and the sample required for each one. For example, one rule of thumb dictates that more than five cases per variable are required for a regression analysis [10].

Before making a final decision on a hypothesis and study design, identify the range of sample sizes that will be required for your research under different conditions. Early identification of a sample size that is prohibitively large will prevent time being wasted designing a study destined to be underpowered. Importantly, heuristics should not be used as the main source of information for sample size calculation. Rules of thumb are rarely congruous with careful sample size calculation [10] and will likely lead to an underpowered study. They should only be used, along with the information gathered through the use of the other techniques recommended in this paper, as a guide to inform the hypothesis and study design.

### Other considerations

#### Be mindful of multiple comparisons

The nature of statistical significance is that one in every 20 hypotheses tested will give a (false) significant result. This should be kept in mind when running multiple tests on the collected data. The hypothesis and appropriate tests should be nominated before the data are collected and only those tests should be performed. There are ways to correct for multiple comparisons [9], however, many argue that this is unnecessary [11]. There is no definitive way to ‘fix’ the problem of multiple tests being performed on a single data set and statisticians continue to argue over the best methodology [12, 13]. Despite its complexity, it is worth considering how multiple comparisons may affect the results, and if there would be a reasonable way to adjust for this. The decision made should be noted and explained in the submitted manuscript.

#### Importance

After reading some introductory literature around sample size calculation it should be possible to derive an estimate to meet the study requirements. If this sample is not feasible, all is not lost. If the study is novel, it may add to the literature regardless of sample size. It may be possible to use pilot data from this preliminary work to compute a sample size calculation for a future study, to incorporate a qualitative component (e.g., interviews, focus groups), for answering a research question, or to inform new research.

#### Post hoc power analysis

This involves calculating the power of the study retrospectively, by using the observed effect size in the data collected to add interpretation to an insignificant result [2]. Hoenig and Heisey [14] detail this concept at length, including the range of associated limitations of such an approach. The well-reported criticisms of post hoc power analysis should cultivate research practice that involves appropriate methodological planning prior to embarking on a project.

## Conclusion

**Fig. 4:**
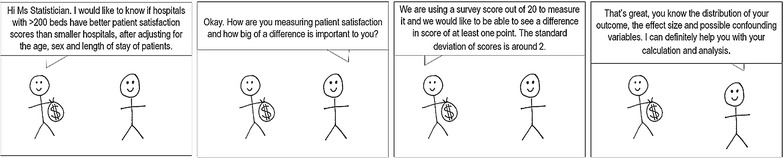
A statistician’s dream

Health services research can be a difficult environment for sample size calculation. However, it is entirely possible that, provided that significance, power, effect size and study design have been appropriately considered, a logical, meaningful and defensible calculation can always be obtained, achieving the situation described in Fig. 4.

## Additional file

10.1186/s13104-016-1893-x Case study. This case study illustrates the steps of a sample size calculation.

## Footnotes

1 Literature summarising an aspect of sample size calculation is included in Table 1, providing a comprehensive mix of different aspects. The list is not exhaustive, and is to be used as a starting point to allow researchers to perform a more targeted search once their sample size problems have become clear. A librarian was consulted to inform a search strategy, which was then refined by the lead author. The resulting literature was reviewed by the lead author to ascertain suitability for inclusion.

## References

[1] Merrifield A, Smith W (2012). Sample size calculations for the design of health studies: a review of key concepts for non-statisticians. NSW Public Health Bull.

[2] Lenth RV (2001). Some practical guidelines for effective sample size determination. Am Stat.

[3] Martinez-Mesa J, Gonzalez-Chica DA, Bastos JL, Bonamigo RR, Duquia RP (2014). Sample size: how many participants do i need in my research?. An Bras Dermatol.

[4] Webb P, Bain C (2011). Essential epidemiology: an introduction for students and health professionals.

[5] Omar RZ, McNally N, Ambler G, Pollock AM (2006). Quality research in healthcare: are researchers getting enough statistical support?. BMC Health Serv Res.

[6] Maxwell SE, Kelley K, Rausch JR (2008). Sample size planning for statistical power and accuracy in parameter estimation. Annu Rev Psychol.

[7] Altman DG, Goodman SN, Schroter S (2002). How statistical expertise is used in medical research. J Am Med Assoc.

[8] Sullivan GM, Feinn R (2012). Using effect size—or why the P value is not enough. J Grad Med Educ.

[9] Walker AM (1986). Reporting the results of epidemiologic studies. Am J Public Health.

[10] Green SB (1991). How many subjects does it take to do a regression analysis. Multivariate Behav Res.

[11] Feise R (2002). Do multiple outcome measures require p-value adjustment?. BMC Med Res Methodol.

[12] Savitz DA, Olshan AF (1998). Describing data requires no adjustment for multiple comparisons: a reply from Savitz and Olshan. Am J Epidemiol.

[13] Savitz DA, Olshan AF (1995). Multiple comparisons and related issues in the interpretation of epidemiologic data. Am J Epidemiol.

[14] Hoenig JM, Heisey DM (2001). The abuse of power: the pervasive fallacy of power calculations for data analysis. Am Stat.

[15] Noordzij M, Tripepi G, Dekker FW, Zoccali C, Tanck MW, Jager KJ (2010). Sample size calculations: basic principles and common pitfalls. Nephrol Dial Transplant.

[16] Vardeman SB, Morris MD (2003). Statistics and ethics: some advice for young statisticians. Am Stat.

[17] Dowd BE (2011). Separated at birth: statisticians, social scientists, and causality in health services research. Health Serv Res.

[18] Dimick JB, Welch HG, Birkmeyer JD (2004). Surgical mortality as an indicator of hospital quality: the problem with small sample size. J Am Med Assoc.

[19] Thomas DC, Siemiatycki J, Dewar R, Robins J, Goldberg M, Armstrong BG (1985). The problem of multiple inference in studies designed to generate hypotheses. Am J Epidemiol.

[20] VanVoorhis CW, Morgan BL (2007). Understanding power and rules of thumb for determining sample sizes. Tutor Quant Methods Psychol.

[21] Van Belle G (2011). Statistical rules of thumb.

[22] Serumaga-Zake PA, Arnab R, editors. A suggested statistical procedure for estimating the minimum sample size required for a complex cross-sectional study. The 7th international multi-conference on society, cybernetics and informatics: IMSCI, 2013 Orlando, Florida, USA; 2013.

[23] Hsieh FY, Bloch DA, Larsen MD (1998). A simple method of sample size calculation for linear and logistic regression. Stat Med.

[24] Alam MK, Rao MB, Cheng F-C (2010). Sample size determination in logistic regression. Sankhya B.

[25] Peduzzi P, Concato J, Kemper E, Holford TR, Feinstein AR (1996). A simulation study of the number of events per variable in logistic regression analysis. J Clin Epidemiol.

[26] Dupont WD, Plummer WD (1998). Power and sample size calculations for studies involving linear regression. Control Clin Trials.

[27] Zhong B (2009). How to calculate sample size in randomized controlled trial?. J Thorac Dis.

[28] Maas CJM, Hox JJ (2005). Sufficient sample sizes for multilevel modeling. Methodology.

[29] Cohen MP (2005). Sample size considerations for multilevel surveys. Int Stat Rev.

[30] Paccagnella O (2011). Sample size and accuracy of estimates in multilevel models: new simulation results. Methodology.

[31] Maas CJM, Hox JJ (2004). Robustness issues in multilevel regression analysis. Stat Neerl.

