# Supplementary material for: When is enough, enough? Understanding and solving your sample size problems in health services research
Source: BMC Res Notes. 2016 Feb 12;9:90. doi: 10.1186/s13104-016-1893-x (PMC4751721; doi:10.1186/s13104-016-1893-x)
Supplement: Supplementary file 1 — 10.1186/s13104-016-1893-x Case study. This case study illustrates the steps of a sample size calculation. [file 13104_2016_1893_MOESM1_ESM.pdf]

Additional file 1. Case study

Scenario: The researcher wants to identify whether hospitals with more than 200 beds perform better than smaller hospitals, using four measures: re-admission rates, mortality, nosocomial infection rates and patient satisfaction.

Step 1: Decide on the primary research question.

Firstly consider the type of tests that might be done for each outcome. A simple t-test could compare the outcome measures between large and small hospitals. Or a regression, or multi-level model, might be used to adjust for other factors. Which outcome is the most important in the research?

In this case patient satisfaction, measured as a survey score out of 20, was deemed most important.

Primary research question: Do hospitals with more than 200 beds have higher patient satisfaction scores after adjusting for the age, sex and length of stay of the participants?

Step 2: Decide study design analysis method (see Table 1 for rules of thumb).

For this example, a linear regression analysis will allow the researcher to identify the impact of hospital size (big/small) on patient satisfaction, whilst adjusting for other predictors.

Step 3: Find inputs for sample size calculation from the literature.

The researcher will need to estimate effect size:

- Decided to use Cohen's *d*, from Sullivan and Feinn [8].
- From the results the mean and standard deviation of outcome (patient satisfaction score out of 20) = 16(2). This could be from the literature or pilot studies.
- The difference in score deemed important = 1. This could be from consultation with experts in the field.

Using equation from Sullivan and Feinn [8] this results in an effect size of 0.5, which is considered medium.

Step 4: Use rules of thumb and calculation from the literature to decide on appropriate sample size.

For this example we used Table 1 in Green [10] to decide a sample size of 81 – see also Lenth [2] and Martinez-Mesa et al. [3].

Solution: For this scenario, with a medium effect size, and four predictor variables, the required sample size is 81. This means the researcher will have to collect 81 patient satisfaction surveys. These would need to be collected (theoretically) from a simple random sample of the population of all patient satisfaction surveys (and so all patients) within the sample frame.

Alternative Solution: If a simple random sample could not be collected the researcher could consider multilevel modelling.
